# Morphometric Magnetic Resonance Imaging Evaluation of Cervical Spinal Canal and Cord in Normal Small-Breed Dogs

**DOI:** 10.3389/fvets.2021.732953

**Published:** 2021-09-29

**Authors:** Namsoon Lee, Jeonghyun Seo, Junghee Yoon

**Affiliations:** ^1^Time Animal Medical Center, Daejeon, South Korea; ^2^College of Veterinary Medicine and the Research Institute for Veterinary Science, Seoul National University, Seoul, South Korea

**Keywords:** cervical spinal canal, spinal cord, MRI, height, width, CSA, small breed dogs

## Abstract

Cervical spinal disease is one of the most common neurological disorders in small-breed dogs. Magnetic resonance imaging (MRI) is a common test for dogs with cervical spinal diseases. However, there is no information on cervical spinal canal and cord using MRI in normal small-breed dogs. Therefore, this study aimed to perform analyses to establish morphologic MRI reference ranges of the cervical spinal canal and cord in normal small-breed dogs. Cervical MRI examinations were taken in 20 client-owned small dogs. The height, width, and cross-sectional area (CSA) of the spinal canal and cord were measured on sagittal and transverse T2-weighted images at each vertebral body level and each intervertebral disk level from C1–C2 to C7 (a total of 12 levels). The height ratio, the width ratio, and the CSA ratio were calculated. The height, width, and CSA of the spinal canal and cord increased as the dog's weight increased (*p* < 0.01), except for that at C1–C2. However, there was no correlation between the body weight and height ratio and the width ratio and CSA ratio at all levels, except for that at C1–C2. Also, there was a negative correlation between the body weight and CSA ratio at C1–C2. There were no statistical differences for the CSA of the spinal canal, the CSA of the spinal cord, and the CSA ratio between nearby levels, except for that at C1–C2. There was no statistical difference between measurements at each same level of the sagittal and transverse images. The results of this study may provide basic and morphometric information for diagnosing and researching cervical spinal diseases in small-breed dogs.

## Introduction

Cervical spinal disease is one of the most common neurological disorders in small-breed dogs. Cervical intervertebral disk disease (IVDD) accounts for ~15% of all intervertebral disk extrusions in small-breed dogs, with breeds such as Dachshund, beagle, and poodle representing 80% of the cases (1–3). Young small- and toy-breed dogs like Maltese, chihuahua, toy poodle, and Yorkshire terrier are overrepresented for atlantoaxial instability (4). Syringomyelia (SM) is also a frequent diagnosis in brachycephalic toy breeds (5).

Previous studies have evaluated the anatomic and quantitative qualities of the cervical spinal canal or spinal cord in normal dogs using radiography, computed tomography (CT), and CT myelography (6–10). However, magnetic resonance imaging (MRI) has become a gold standard to diagnose neurological diseases, and its availability has been increasing for dogs with cervical spinal diseases (11). There have been a few previous studies on the morphometric or quantitative analysis of the cervical spinal region using MRI, but these only focused on specific large breeds and diseases (12–15). One study provided the normal heights of the spinal cord and canal in small to large dogs, but only the sagittal height was measured and the report did not investigate the cervical region (16). There was a comparative study between small-breed dogs and specific large-breed dogs, but it did not evaluate the entire cervical spine and was not a study in normal small-breed dogs (17).

To the authors' knowledge, there is no information on the cervical spinal canal and cord using MRI in normal small-breed dogs. Thus, in this study, we aimed to perform analyses to establish MRI reference ranges for the height, width, and cross-sectional area (CSA) of the cervical spinal canal and cord and the ratio of spinal cord size to canal in normal small-breed dogs.

## Materials and Methods

The study protocol was approved by the Seoul National University Institutional Animal Care and Use Committee (approval no. SNU-210125-1-1). Cervical MRI examinations were taken in 20 client-owned small dogs presented to the Time Animal Medical Center from March 2020 to June 2021. All dogs were healthy based on physical, complete blood cell counts, serum biochemistry, and cerebrospinal fluid (CSF) analyses. None of the dogs demonstrated cervical neurological symptoms or spinal cord or disk abnormalities on cervical MRI and had no microchip in the cervical area. The dogs were normal, except for some with vision loss or presumptively idiopathic seizure. MRI scans were performed with a 1.5-T system (Intera, Philips Healthcare, Eindhoven, Netherlands) under general anesthesia. The anesthesia was induced in each dog with intravenous propofol (Provive 1%, Myungmoon Pharm. Co., Seoul, South Korea) at a dose of 6 mg/kg and maintained with isoflurane (Ifran, Hana Pharm., Gyeonggi-do, South Korea) and oxygen. The dogs were positioned in ventral recumbency, with the forelimbs pulled caudally and a straight spinal alignment on the eight-channel knee coil. Sagittal and transverse T2-weighted images, with a repetition time of 3,000–4,000 ms, echo time of 100 ms, slice thickness of 2.0 mm, and gap of 0.2 mm with the phase-encoding direction being anterior–posterior, were obtained from each dog for measurements. Dorsal T2-weighted and transverse T1-weighted images were also obtained for reference of the surrounding structures.

### Measurements and Analysis of MR Images

**Figure 1:**
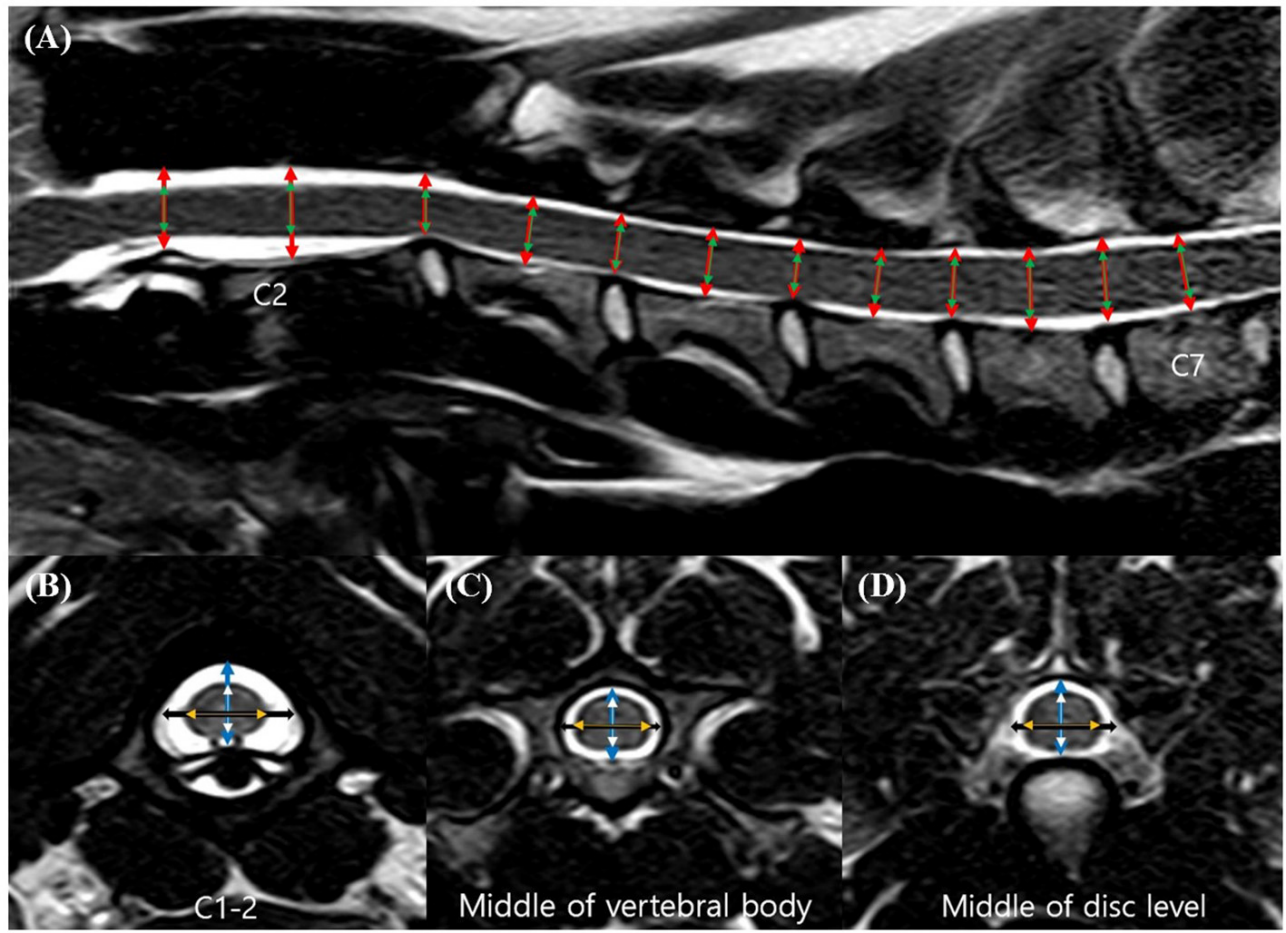
Sagittal **(A)** and transverse **(B–D)** T2-weighted magnetic resonance (MR) images. Sagittal location of the measurements: the height of the spinal canal (*red arrows*) and the height of the spinal cord (*green arrows*). Transverse location of the measurements: the height of the spinal canal (*blue arrows*), the height of the spinal cord (*white arrows*), the width of the spinal canal (*black arrows*), and the width of the spinal cord (*orange arrows*).

Magnetic resonance images were analyzed *via* picture archiving and the use of a communication system (PACSPLUS, Medical Standard, Gyeonggi-do, South Korea). The images were independently analyzed by two observers (NL and JS). Measurements followed the methods described in previous reports of dogs and humans (12, 14–16, 18). On the midsagittal T2-weighted images, the heights of the spinal canal (CSF column) and cord were measured at the middle aspect of each vertebral body level from the second cervical vertebrae (C2) to the seventh cervical vertebrae (C7) and at the middle aspect of each intervertebral disk level from the first to the second cervical intervertebral disk space (C1–C2) to the sixth to the seventh cervical intervertebral disk space (C6–C7; a total of 12 levels) (Figure 1A). The heights were measured on a line drawn perpendicular to the spinal cord. On the transverse T2-weighted images, the height and the width of the spinal canal and cord were measured at the same level of the lines used for the sagittal measurements, as described earlier. The heights of the spinal canal and cord were measured on a line drawn from the midpoint of the vertebral body to the midpoint of the corresponding spinous process. The ratio of the height of the spinal cord to the canal was calculated by dividing the height of the spinal cord by the height of the spinal canal (the height ratio). The widths of the spinal canal and cord were also measured on a line drawn perpendicular to the line of the height (Figures 1B–D). The ratio of the width of the spinal cord to the canal was calculated by dividing the width of the spinal cord by the width of the spinal canal (the width ratio). By dividing the height by the width (height-to-width ratio), an approximate roundness index of the spinal canal and cord was obtained for each region.

**Figure 2:**
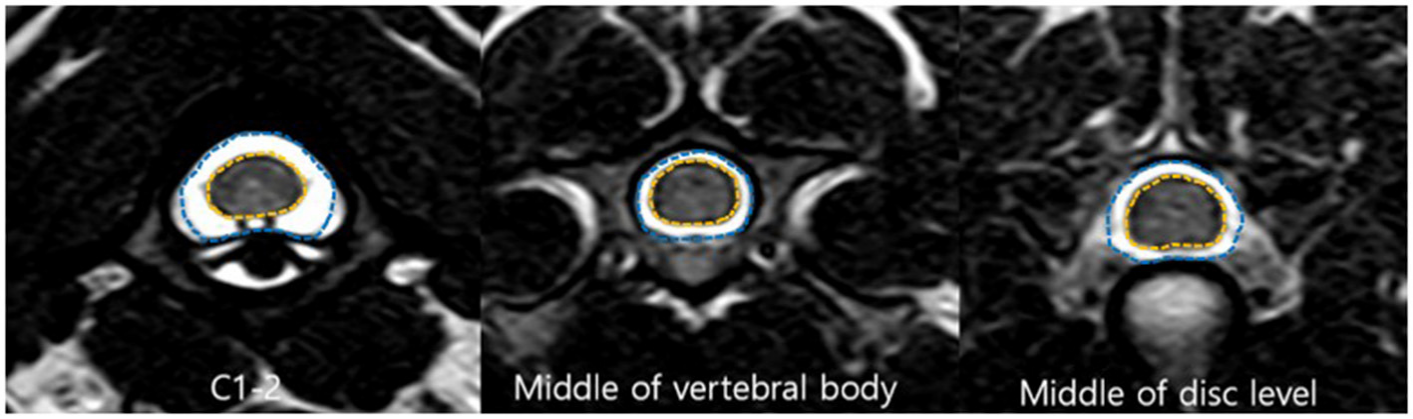
Transverse T2-weighted magnetic resonance (MR) images. Cross-sectional area (CSA) of the spinal canal (*blue line*) and the spinal cord (*orange line*).

The CSAs of the spinal canal and cord were measured in the same transverse images with manually traced regions of interest (Figure 2). The proportion of the spinal canal occupied by the spinal cord was determined by dividing the CSA of the spinal cord by the CSA of the spinal canal (the CSA ratio).

### Statistical Analysis

Statistical analyses of the data were performed using SPSS software (IBM SPSS Statistics 22, IBM Corporation, Armonk, NY, USA). Normally distributed data were presented as the mean ± standard deviation (SD). Pearson's correlations or Kendall's tau was used to identify the correlations between the measurements and body weight. A linear relationship between the CSA ratio of the spinal cord to the spinal canal at C1–C2 and body weight was verified by regression analysis and scatter plots. The Mann–Whitney *U*-test was used to compare the values between dogs ≤5 kg and dogs >5 kg at the CSA ratio of the spinal cord to the spinal canal at C1–C2. One-way analysis of variance (ANOVA) or Kruskal–Wallis ANOVA on ranks was used for comparisons between the measurements in all levels, and Scheffe's *post-hoc* tests were used to correct for multiple comparisons. A *t*-test was used for comparisons between measurements at each same level of the sagittal and transverse images. Intra-observer and inter-observer reproducibility was assessed using the intraclass correlation coefficient (ICC). A value close to 1 indicates excellent agreement. A *p*-value < 0.05 was considered statistically significant.

## Results

The breeds of the dogs included in the study were Maltese (six), Pomeranian (one), toy poodle (one), Spitz (one), mixed breed (nine), and Beagles (two). Eleven dogs were male and nine were female. The mean age was 3.60 years (range, 1.6–8.0 years) and the mean body weight was 8.23 kg (range, 2.6–15.0 kg).

The measurements for each level (12 levels) and the ratio are summarized in Tables 1–5.

**Table 1:** Heights of the spinal canal and cord measured on sagittal images and ratio of the height of the spinal cord to the canal.

**Level**	**Canal (mm)**	**Cord (mm)**	**Cord-to-canal ratio**
C1–C2	8.45 ± 1.06	5.48 ± 0.70	0.65 ± 0.06
C2	9.47 ± 1.50	5.72 ± 0.74	0.61 ± 0.05
C2–C3	7.00 ± 1.08	4.93 ± 0.77	0.71 ± 0.04
C3	7.38 ± 0.89	5.39 ± 0.78	0.73 ± 0.04
C3–C4	6.66 ± 0.91	4.97 ± 0.71	0.75 ± 0.03
C4	7.39 ± 0.88	5.49 ± 0.76	0.74 ± 0.02
C4–C5	7.00 ± 0.87	5.43 ± 0.71	0.78 ± 0.03
C5	7.98 ± 0.93	5.97 ± 0.81	0.75 ± 0.02
C5–C6	8.06 ± 1.00	6.18 ± 0.81	0.77 ± 0.03
C6	8.69 ± 1.00	6.48 ± 0.82	0.74 ± 0.03
C6–C7	8.24 ± 0.98	6.32 ± 0.82	0.77 ± 0.03
C7	8.02 ± 0.88	5.96 ± 0.74	0.74 ± 0.04

*Data are expressed as the mean ± standard deviation*.

*C1, first cervical vertebra; C1–C2, first to second cervical intervertebral disk space; C2, second cervical vertebra; C2–C3, second to third cervical intervertebral disk space; C3, third cervical vertebra; C3–C4, third to fourth cervical intervertebral disk space; C4, fourth cervical vertebra; C4–C5, fourth to fifth cervical intervertebral disk space; C5, fifth cervical vertebra; C5–C6, fifth to sixth cervical intervertebral disk space; C6, sixth cervical vertebra; C6–C7, sixth to seventh cervical intervertebral disk space; C7, seventh cervical vertebra*.

**Table 2:** Heights of the spinal canal and cord measured on transverse images and ratio of the height of the spinal cord to the canal.

**Level**	**Canal (mm)**	**Cord (mm)**	**Cord-to-canal ratio**
C1–C2	8.49 ± 1.07	5.54 ± 0.68	0.67 ± 0.07
C2	9.56 ± 1.49	5.74 ± 0.78	0.61 ± 0.05
C2–C3	7.06 ± 1.09	5.03 ± 0.78	0.71 ± 0.04
C3	7.39 ± 0.91	5.42 ± 0.82	0.73 ± 0.04
C3–C4	6.75 ± 0.92	5.10 ± 0.71	0.76 ± 0.03
C4	7.40 ± 0.87	5.55 ± 0.80	0.75 ± 0.03
C4–C5	7.10 ± 0.85	5.55 ± 0.73	0.78 ± 0.03
C5	7.97 ± 0.94	6.06 ± 0.84	0.76 ± 0.03
C5–C6	8.11 ± 0.98	6.26 ± 0.83	0.77 ± 0.03
C6	8.68 ± 1.01	6.54 ± 0.84	0.75 ± 0.03
C6–C7	8.25 ± 0.98	6.34 ± 0.84	0.77 ± 0.03
C7	8.03 ± 0.91	6.00 ± 0.75	0.75 ± 0.04

*Data are expressed as the mean ± standard deviation. Abbreviations are the same as those in Table 1*.

**Table 3:** Widths of the spinal canal and cord measured on transverse images and ratio of the width of the spinal cord to the canal.

**Level**	**Canal (mm)**	**Cord (mm)**	**Cord-to-canal ratio**
C1–C2	12.24 ± 1.45	8.64 ± 1.00	0.71 ± 0.06
C2	9.50 ± 1.27	7.10 ± 1.29	0.75 ± 0.05
C2–C3	10.06 ± 0.86	7.54 ± 1.03	0.75 ± 0.06
C3	9.63 ± 1.01	7.13 ± 1.12	0.74 ± 0.06
C3–C4	10.30 ± 0.78	7.63 ± 0.94	0.74 ± 0.06
C4	9.79 ± 1.00	7.30 ± 1.04	0.74 ± 0.05
C4–C5	10.30 ± 0.73	7.65 ± 0.96	0.74 ± 0.06
C5	10.44 ± 0.96	7.86 ± 1.07	0.75 ± 0.06
C5–C6	10.90 ± 0.81	8.23 ± 1.09	0.75 ± 0.06
C6	11.05 ± 1.08	8.28 ± 1.10	0.74 ± 0.04
C6–C7	10.92 ± 0.97	8.09 ± 1.17	0.74 ± 0.07
C7	10.30 ± 0.87	7.67 ± 1.07	0.74 ± 0.06

*Data are expressed as the mean ± standard deviation. Abbreviations are the same as those in Table 1*.

**Table 4:** Height-to-width ratio of the spinal canal and cord (roundness index).

**Level**	**Canal**	**Cord**
C1–C2	0.70 ± 0.08	0.65 ± 0.07
C2	1.00 ± 0.07	0.82 ± 0.11
C2–C3	0.70 ± 0.07	0.67 ± 0.08
C3	0.77 ± 0.05	0.76 ± 0.05
C3–C4	0.66 ± 0.07	0.67 ± 0.06
C4	0.76 ± 0.05	0.76 ± 0.04
C4–C5	0.69 ± 0.07	0.72 ± 0.06
C5	0.76 ± 0.05	0.77 ± 0.05
C5–C6	0.74 ± 0.07	0.76 ± 0.07
C6	0.79 ± 0.04	0.80 ± 0.06
C6–C7	0.76 ± 0.09	0.79 ± 0.09
C7	0.78 ± 0.07	0.79 ± 0.08

*Data are expressed as the mean ± standard deviation. Abbreviations are the same as those in Table 1*.

**Table 5:** Cross-sectional area (CSA) of the spinal canal and cord measured on transverse images and ratio of the CSA of the spinal cord to the canal.

**Level**	**Canal (mm^**2**^)**	**Cord (mm^**2**^)**	**Cord-to-canal ratio**
C1–C2	91.64 ± 20.97	39.27 ± 8.00	0.44 ± 0.06
C2	72.86 ± 19.38	33.74 ± 9.40	0.47 ± 0.04
C2–C3	57.86 ± 12.47	31.62 ± 8.24	0.54 ± 0.05
C3	58.21 ± 12.55	33.30 ± 9.44	0.57 ± 0.06
C3–C4	57.70 ± 11.31	33.19 ± 8.66	0.57 ± 0.06
C4	59.00 ± 11.46	34.75 ± 9.37	0.58 ± 0.06
C4–C5	60.83 ± 10.91	36.12 ± 9.20	0.59 ± 0.06
C5	67.07 ± 12.85	40.30 ± 10.69	0.59 ± 0.06
C5–C6	71.43 ± 12.59	42.94 ± 10.43	0.60 ± 0.06
C6	76.24 ± 14.78	44.88 ± 10.92	0.58 ± 0.05
C6–C7	73.78 ± 13.19	43.70 ± 10.22	0.59 ± 0.05
C7	66.26 ± 12.07	37.85 ± 9.13	0.57 ± 0.05

*Data are expressed as the mean ± standard deviation. Abbreviations are the same as those in Table 1*.

C1–C2 showed the largest width of the spinal canal and cord and CSA of the spinal canal compared to those of other levels; thus, their roundness indices were the smallest, as well as the CSA ratio. At C2, the height of the spinal canal was the largest compared to that of the other levels, and given that the roundness index of the spinal canal was the largest, the shape was almost round (almost 1.0). The CSA ratio at C2 was the second smallest after C1. The CSA of the spinal cord at C2–C3 was the smallest and that at C6 was the largest compared to that of the other levels. The CSA ratio at the disk level was smaller than those at nearby vertebral body levels, but there was no statistical significance. Moreover, there were no statistical differences in the CSA of the spinal canal, the CSA of the spinal cord, and the CSA ratio between nearby levels, except for those of C1–C2.

**Figure 3:**
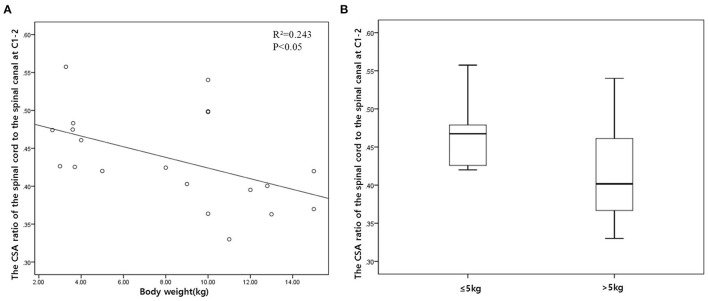
**(A)** Scatter plot and linear regression for the cross-sectional area (CSA) ratio of the spinal cord to the spinal canal (the CSA ratio) in relation to body weight at C1–C2. **(B)** Box plot of the CSA ratio at C1–C2 compared by dividing the dogs into two groups: dogs ≤5 kg (eight dogs) and dogs >5 kg (12 dogs).

The height, width, and CSA of the spinal canal and cord increased as the dog's weight increased (*p* < 0.01), except for those of C1–C2. However, there was no correlation between the body weight and height ratio and the width ratio and CSA ratio at all levels, except for those of C1–C2. There was a negative correlation between the body weight and the CSA ratio at C1–C2 (Figure 3A). When the CSA ratios at C1–C2 were compared by dividing the dogs into two groups, dogs ≤5 kg (eight dogs) and dogs >5 kg (12 dogs), the CSA ratios of the dogs ≤5 kg were greater than those of the dogs >5 kg (*p* = 0.047) (Figure 3B). There was no statistical difference between the measurements at each same level of the sagittal and transverse images. Agreement between the two observers regarding the measurements was excellent (intra-observer = 0.93–0.99; inter-observer = 0.92–0.98).

## Discussion

In previous studies, the ratio of the spinal cord area to the spinal canal area was greater in small dogs compared to that of large dogs (8, 9, 17). It was proposed that a relatively narrower subarachnoid space in the cervical region can predispose neck pain easily in small dogs with IVDD (8). However, a comparative study between small-breed dogs and specific large-breed dogs for cervical spondylomyelopathy (CSM) proposed that this may not play a primary role in the pathogenesis of CSM (17). Further studies on the clear pathophysiology between the relative spinal cord to canal size and neurological symptoms are needed; however, it is important to establish normal values in normal small-breed dogs prior to that.

In the present study, the height, width, and CSA of the spinal canal and cord showed a tendency to increase as the body weight increases, similar to that in previous studies (9, 17). However, there was no correlation between the body weight and the relative spinal cord size to canal (the CSA ratio) at all levels, except for that at C1–C2 because they were the same group of small dogs and, thus, there will be no significant difference between them. Further studies are required to investigate and compare normal small- and large-breed dogs using MRI to confirm this tendency in the cervical region.

Dogs ≤5 kg showed a relatively larger cord size at C1–C2 than did dogs >5 kg. Although dogs with an apparent anomaly of the cranio-cervical junction (CCJ) and SM were excluded in this study, most of the dogs ≤5 kg were toy-breed dogs that are predisposed to these conditions (19). In the present study, C1–C2 and C2 showed different aspects from the rest of the spinal canal and cord in terms of size, shape, and relative size. Morphologies of the first cervical vertebrae (C1) and C2 are quite distinct from that of the other cervical vertebrae, and these, as well as the CSF in these regions, are influenced by any abnormal shape of the CCJ (20, 21); thus, it cannot be excluded that the distinct morphology of the region may have influenced this result or that this result may be a factor in the frequent occurrence of diseases in this region in toy-breed dogs. In the study by Christen et al. on the quantification of CSF flow in dogs, it was proposed that the head position and angle may influence the CSF flow in this region (22). Therefore, future studies are needed to investigate this region further.

To the authors' knowledge, there are no well-defined guidelines to grade the severity of spinal compression on MRI. In previous studies and in the veterinary literature, the spinal cord compression ratio was obtained by measuring the percentage of the CSA of the spinal canal that is occupied by the compression material or the ratio of the CSA of the spinal cord at the point of maximal compression to the CSA of the closest spinal cord without compression (23–26). However, in order to use these methods, there must be certain prerequisites, that is, a normal range for the normal spinal canal and equal CSA of the nearby spinal cord. Given that there was no statistical difference in the CSA of the spinal cord between the nearby levels, the commonly used methods described in previous studies can be used to grade the severity of spinal compression on MRI. However, all measurements of the spinal cord at the sixth cervical vertebrae (C6) and at C6–C7 were larger than those of the other levels, except for that of C1–C2, which could have been due to the cervical intumescence located from C6 to C7 (20, 26). Therefore, these should not be mistaken for pathologic cord swelling on MRI (26). Since the cervical intumescence might affect the measurements, it is recommended to measure at C6 rather than at C7 when using the CSA of the spinal cord at nearby levels to evaluate the severity of spinal compression at C6–C7.

T2-weighted sagittal images are routinely acquired prior to planning transverse images as a guide. However, it has been reported that the evaluation of sagittal images in IVDD was not 100% accurate (27). Given that transverse images provide a more accurate representation of anatomical structures, landmarks are more easily identified in transverse images (11). It means that there could be a difference between the sagittal and the transverse measurements. We measured the height of the spinal cord and canal at each same level of the sagittal and transverse images and found no statistical difference between them. Given that it is extremely important to achieve a straight spinal alignment to allow comparisons of multiple intervertebral sites on sagittal images (11), MRI examination was performed in the dogs, maintaining a straight spinal alignment as much as possible and performing all measurements on the midsagittal T2-weighted images in the present study. If these conditions are satisfied, measurements on the T2-weighted images may be as useful as those on transverse images.

Most measurements were in near-perfect agreement between intra- and inter-observers at the vertebral body levels, except for that of the transverse images at the disk levels. Given that it was difficult to discriminate the margin of the CSF due to epidural fat in some images (26), we measured it with reference to the T1-weighted images, and the agreement was excellent.

A limitation of this study was that the number of dogs was small; therefore, we had to use a non-parametric test in some statistical analyses. Another limitation is that a relatively large number of mixed-breed dogs and a relatively low variety of breeds were included in this study. Further studies with larger groups and more representative small-breed dogs are needed. There were also fewer dogs ≤5 kg that showed a larger tendency of relative cord size at C1–C2. Given that there are significant morphological differences in the cervical region like the vertebral body and disk length between dog breeds (28), it cannot be excluded that differences in the relative cord size at C1–C2 may be due to the inherently variable anatomic shapes in this region. Therefore, future studies will also be needed to investigate this region in toy-breed dogs. Despite the above limitations, the results of this study may provide basic and morphometric information for diagnosing and researching cervical spinal diseases in small-breed dogs.

In conclusion, this study provides the height of the spinal canal and cord on the midsagittal T2-weighted images, the height, width, and CSA of the spinal canal and cord on the transverse T2-weighted images, and the height ratio, width ratio, height-to-width ratio, and CSA ratio from C1–C2 to C7 in normal small-breed dogs. In particular, the CSA ratio is useful for evaluating the degree of spinal cord compression, and dogs ≤5 kg that showed a relatively larger cord size at C1–C2 need future studies on the relationship between the morphological characteristics of the spinal cord at C1–C2 and common diseases in this region. These measurements may serve as a morphometric baseline for diagnosing and researching cervical spinal diseases in small-breed dogs.

## Data Availability Statement

The original contributions presented in the study are included in the article/supplementary material, further inquiries can be directed to the corresponding author/s.

## Ethics Statement

The animal study was reviewed and approved by Seoul National University Institutional Animal Care and Use Committee (Approval number SNU-210125-1-1). Written informed consent was obtained from the owners for the participation of their animals in this study.

## Author Contributions

NL and JY contributed to the conception and design of the study. NL and JS organized the database and wrote sections of the manuscript. NL performed the statistical analysis and wrote the first draft of the manuscript. All authors contributed to manuscript revision and read and approved the submitted version.

## Conflict of Interest

The authors declare that the research was conducted in the absence of any commercial or financial relationships that could be construed as a potential conflict of interest.

## Publisher's Note

All claims expressed in this article are solely those of the authors and do not necessarily represent those of their affiliated organizations, or those of the publisher, the editors and the reviewers. Any product that may be evaluated in this article, or claim that may be made by its manufacturer, is not guaranteed or endorsed by the publisher.
